# [Expression of Concern] lncRNA HOTTIP facilitates osteosarcoma cell migration, invasion and epithelial-mesenchymal transition by forming a positive feedback loop with c-Myc

**DOI:** 10.3892/ol.2026.15670

**Published:** 2026-05-26

**Authors:** Yang Tang, Fang Ji

Oncol Lett 18:1649–1656, 2019; DOI: 10.3892/ol.2019.10463

Following the publication of the above paper, it has been drawn to the Editor's attention by a concerned reader that, regarding the cell migration assay experiments shown in Fig. 6B on p. 1654, the ‘si-NC pcDNA3.1’ and ‘si-HOTTIP 1+2, 3.1-c-myc-1’ data panels contained an overlapping section, suggesting that these data panels, which were intended to show the results from differently performed experiments, suggesting that these data were derived from the same original source

The authors have been contacted by the Editorial Office to offer an explanation for this apparent anomaly in the presentation of the data in this paper, and we are awaiting their response. Owing to the fact that the Editorial Office has been made aware of this potential issue surrounding the scientific integrity of this paper, we are issuing an Expression of Concern to notify readers of this potential problem while the Editorial Office continues to investigate this matter further.

